# Compliance with the 24-h movement guidelines and the relationship with anthropometry in Finnish preschoolers: the DAGIS study

**DOI:** 10.1186/s12889-019-7967-7

**Published:** 2019-12-03

**Authors:** Marja H. Leppänen, Carola Ray, Heini Wennman, Christina Alexandrou, Katri Sääksjärvi, Leena Koivusilta, Maijaliisa Erkkola, Eva Roos

**Affiliations:** 1 Folkhälsan Research Center, Helsinki, Finland; 2 Department of Food and Nutrition, University of Helsinki, Helsinki, Finland; 3 Finnish Institute for Health and Welfare, Helsinki, Finland; 4 Department of Biosciences and Nutrition, Karolinska Institutet, Huddinge, Sweden; 5 Faculty of Educational Sciences, University of Helsinki, Helsinki, Finland; 6 Department of Social Research, University of Turku, Turku, Finland; 7 Department of Public Health, Clinicum, University of Helsinki, Helsinki, Finland

**Keywords:** Physical activity, Screen time, Sleep, Body mass index, Waist circumference, 24-h guidelines, Children

## Abstract

**Background:**

Recent 24-h movement guidelines for the early years established recommendations for physical activity (PA), screen time (ST), and sleep. To date, few studies have focused on compliance with meeting the guidelines and their associations with health outcomes. Thus, we aimed to investigate: 1) compliance with the 24-h movement guidelines, and 2) associations between compliance and anthropometry in Finnish preschoolers.

**Methods:**

We utilized DAGIS survey data that were collected in 2015–2016 (*N* = 864). PA was assessed 24 h/day over 7 days using a waist-worn ActiGraph wGT3X-BT accelerometer. ST and sleep were reported by the parents during the same 7 days. Anthropometry was assessed using body mass index (BMI, kg/m^2^) and waist circumference (WC, cm). Children were classified as meeting the guidelines if they averaged ≥180 min/day of PA, which consisted of ≥60 min of moderate-to-vigorous intensity; ≤60 min/day of ST; and 10–13 h/day of sleep. In total, 778 children (51% boys, mean age: 4.7 ± 0.9 years) were included in the study. The compliance with meeting the 24-h movement guidelines was calculated for each behavior separately and in combinations. Adjusted linear regression analyses were applied to examine associations of compliance with BMI and WC.

**Results:**

Children were physically active on average 390 (±46.2) min/day and spent 86 (±25.5) min/day in moderate-to-vigorous PA. They spent 76 (±37.4) min/day on ST and had on average 10:21 (±0:33) h:min/day of sleep. The compliance rate in meeting all three movement guidelines overall was 24%. The highest compliance rate was found for PA (85%), followed by sleep (76%) and ST (35%). Meeting guidelines separately for PA or sleep, or for both, were associated with lower WC (PA: B = -1.37, *p* < 0.001; Sleep: B = -0.72, *p* = 0.009; PA + Sleep: B = -1.03, *p* < 0.001). In addition, meeting guidelines for sleep or for both PA and sleep were associated with lower BMI (Sleep: B = -0.26, *p* = 0.027; PA + Sleep: B = -0.30, *p* = 0.007). There were no significant associations found regarding ST.

**Conclusions:**

Meeting recommendations for PA and sleep may have an important role in supporting a healthy weight status in young children. However, there is still a need to improve compliance with the 24-h movement guidelines, especially for ST.

## Background

Traditionally, research has focused on investigating associations of physical activity (PA), sedentary time, and sleep with different health outcomes separately, showing evidence of health benefits [1–3]. However, because more recent studies have revealed that these movement behaviors may interact with each other [4, 5], there has been growing interest towards an integrated approach to movement behavior. Considering these movement behaviors, PA, sedentary time, and sleep, there were 24-h movement guidelines established for children under 5 years [6, 7]. In accordance with the guidelines, a healthy 24-h day should include: 1) at least 180 min of total PA (TPA), of which at least 60 min is moderate-to-vigorous intensity (MVPA), 2) no more than 1 h of screen time (ST), and 3) 10–13 h of sleep [6]. Previous studies in Canada [8], Australia [9], and Belgium [10] have reported that only 5.6–14.9% of preschool children adhered to the 24-h movement guidelines. The majority of children complied with the PA and sleep guidelines in Canada and Australia [8, 9], whereas the highest rates in Belgium were meeting the guidelines in sleep and ST [10]. Methodological differences, for instance in assessing PA, explain partly the somewhat different results. Thus, there is a need to confirm the results in different study populations.

To date, the associations of complying with the 24-h movement guidelines and health outcomes in preschool children have been studied little. The Australian research group [9] reported that meeting all three of the 24-h movement guidelines was associated with a better performance in social cognition testing. The Canadian research group [8], however, found no association between compliance of the 24-h movement guidelines with body mass index (BMI). Because obesity is currently a major health concern worldwide [11], it is essential to further study associations between compliance with 24-h movement behaviour and adiposity using multiple adiposity indicators. Furthermore, there are no previous studies investigating the associations in European preschool children.

With the release of the 24-h movement guidelines for the early years [6, 7], there is a need to investigate the proportion of children meeting the guidelines and whether the compliance is associated with health outcomes. Such knowledge would help to target health promotion actions to the children and their families that need it the most. The aims of our study were to examine: 1) compliance with the individual and combined 24-h movement guidelines and 2) associations between compliance with both BMI and waist circumference (WC) in Finnish preschool children.

## Methods

### Participants and study design

The present study utilizes data from the DAGIS study (the Increased Health and Wellbeing in Preschools) that aimed to diminish socioeconomic differences in preschool children’s energy balance-related behaviors [12]. The study was conducted in early childhood education and care centers (ECEC) in southern and western Finland in 2015–2016. The eligibility criteria for the study were: 1) having at least one group consisting of 3–6-year-old children, 2) providing early education only during the daytime, 3) being Finnish or Swedish speaking (official languages of Finland), and 4) charging income-dependent fees. In total, 864 children (25% of the invited children) and their families from 66 ECECs (43% of the invited ECECs) in 8 municipalities agreed to participate in the study. In the present study, complete data on PA, ST, and sleep in *overall* (including ≥3 weekdays and ≥ 1 weekend days), *weekdays* (including ≥3 weekdays), or *weekend days* (including ≥1 weekend days) were available for 399 boys and 379 girls. Guardians gave their written informed consent. The study was approved by the University of Helsinki Ethical Review Board in the Humanities and Social and Behavioral Sciences in February 2015 (#6/2015).

### Physical activity

PA was measured using a waist-worn ActiGraph wGT3X-BT accelerometer (Pensacola, FL, USA) for 7 days, 24 h per day. Parents reported in a diary the non-wearing hours of the accelerometers (e.g., during water-based activities). A 15-s epoch length was used, and periods of ≥10 min of consecutive zeros were regarded as non-wearing time [13]. A valid day was defined as ≥600 min of awake wearing time. The time spent in different PA intensities was calculated using Butte’s cut-points [14]. Overall PA was calculated as: [(mean PA on weekdays × 5) + (mean PA on weekend days × 2)] / 7. To calculate the proportion of children complying with the PA guideline separately for overall, weekdays, and weekend days, PA was dichotomized into 0 (< 180 min/day of TPA, of which at least 60 min was MVPA) and 1 (≥180 min/day of TPA, of which at least 60 min was MVPA).

### Screen time

Guardians reported ST in a 7-day sedentary behavior diary. The diary was based on a previously validated diary [15], and it was further translated and modified into the Finnish context. Parents were asked to assess the frequency and time (hours/minutes) that their child spent each day: (1) watching TV, (2) watching DVDs or videos, (3) using tablets or smartphones, and (4) using computers or playing computer games. ST is a composition variable of all of the above-mentioned types of activities. Overall ST was calculated as follows: [(mean ST on weekdays × 5) + (mean ST on weekend days × 2)] / 7. To calculate the proportion of children complying with the ST guideline separately for overall, weekdays, and weekend days, ST was dichotomized into 0 (> 60 min of ST/day) and 1 (≤60 min of ST/day).

### Sleep

Using the same 7-day sedentary behavior diary, guardians were asked to report their child’s bedtime and wake-up time. Sleep duration was calculated by subtracting bedtime from wake-up time. Overall sleep duration was calculated as follows: [(mean sleep duration on weekdays × 5) + (mean sleep duration on weekend days × 2)] / 7. To calculate the proportion of children complying with the sleep guideline separately for overall, weekdays, and weekend days, sleep was dichotomized into 0 (< 10 or > 13 h of sleep/day) and 1 (10–13 h of sleep/day).

### Anthropometry

Weight, height, and WC were measured by trained researchers. Body weight was measured to the nearest 0.01 kg with portable bench scales (CAS PB-100/200). Body height was measured to the nearest 0.1 cm with stadiometers (SECA 217). BMI was calculated as body weight (kg) / height^2^ (m), and further, BMI standard deviation score (BMI-SDS) was computed by the national references [16]. Being overweight was defined using the age- and gender-specific BMI cut-offs of the International Obesity Task Force criteria [17]. WC was measured over one layer of clothing twice to the nearest 0.1 cm with measuring tapes (SECA 201), and the mean of these values was calculated. Waist was defined as the midpoint between the top of the iliac crest and the lower margin of the last palpable rib.

### Statistical analysis

Descriptive information is given as arithmetic means and standard deviations (SD) or frequencies and percentages (%). Children were categorized as meeting or not meeting: 1) individual guidelines, 2) combinations of any two guidelines, or 3) all three guidelines. Using multiple linear regression, associations with BMI and WC were assessed for: 1) individual guidelines, 2) combinations of any two guidelines, 3) all three guidelines, and 4) the number of guidelines met. Each model was adjusted for the child’s age and gender (girl/boy), season of conducting the research (categorized as September–October, November–December, or January–April), and the highest education level in the household [categorized as <bachelor’s degree (i.e., comprehensive, vocation, or high school), bachelor’s degree (i.e., bachelor’s degree or college), or > bachelor’s degree (i.e., master’s degree or licentiate/doctor)]. The models, including PA, were additionally adjusted for awake wearing time of the accelerometer, and the models, including WC were additionally adjusted for the child’s height.

All statistical tests were conducted using a two-sided 5% level of significance and performed using SPSS Statistics 25 (IBM, Armonk, NY, USA).

## Results

Out of the 864 participating children, 778 (91.2%) had valid PA, ST, and sleep data in at least one of the three periods (i.e., overall, weekdays, or weekend days). Of those children 721 (92.7%) had valid data overall, 727 (93.4%) on weekdays, and 759 (97.6%) on weekend days.

Table 1 describes the characteristics of the 778 children subdivided by gender. Children’s TPA on average was 391 (±46.2) min/day and they spent 86 (±25.5) min/day in MVPA. Furthermore, they spent on average 76 (±37.4) min/day on ST and had 10:21 (±0:33) h:min/day of sleep. Boys were taller, heavier, had a higher BMI, and spent more time in TPA and MVPA compared to girls (*p* < 0.05).

**Table 1:** Descriptive characteristics of children

	All		Boys		Girls		*p*^d^
	N	Mean ± SD	N	Mean ± SD	N	Mean ± SD	
Age (years)	778	4.7 ± 0.9	399	4.8 ± 0.9	379	4.7 ± 0.9	0.31
Height (cm)	739	109.6 ± 7.8	372	110.5 ± 7.9	369	108.6 ± 7.7	**0.001**
Weight (kg)	778	19.1 ± 3.5	371	19.5 ± 3.5	366	18.8 ± 3.4	**0.005**
BMI (kg/m^2^)	738	21.7 ± 3.5	372	22.3 ± 3.7	366	21.1 ± 3.2	**< 0.001**
BMI-SDS^a^ (kg/m^2^)	738	−0.06 ± 1.0	372	− 0.05 ± 1.0	366	−0.06 ± 1.0	0.94
Overweight or obese^b^ (N, %)	738	85 (11.5)	372	39 (10.5)	366	46 (12.6)	0.45
Waist circumference (cm)	738	53.7 ± 4.0	372	53.9 ± 3.7	366	53.4 ± 4.2	0.076
Parental education level^c^ (N, %)	774		399		375		0.18
< Bachelor’s degree		164 (21.2)		85 (21.3)		79 (21.1)	
Bachelor’s degree		329 (42.5)		158 (39.6)		171 (45.6)	
> Bachelor’s degree		281 (36.3)		156 (39.1)		125 (33.3)	
Awake wearing time of the accelerometer (min/day)							
Overall	764	774.0 ± 35.4	395	774.8 ± 35.5	396	773.2 ± 35.2	0.54
Weekdays	750	776.4 ± 38.4	386	777.0 ± 38.4	364	775.8 ± 38.4	0.67
Weekend days	770	768.6 ± 51.1	394	770.6 ± 50.9	376	766.5 ± 51.3	0.27
Total physical activity (min/day)							
Overall	764	390.5 ± 46.2	395	395.6 ± 46.0	369	385.1 ± 45.9	**0.002**
Weekdays	750	395.3 ± 48.9	386	401.1 ± 48.9	364	389.1 ± 48.3	**0.001**
Weekend days	770	379.3 ± 60.2	394	383.2 ± 60.5	376	375.1 ± 59.7	0.063
Moderate-to-vigorous physical activity (min/day)							
Overall	764	86.0 ± 25.5	395	92.0 ± 25.6	369	79.5 ± 23.7	**< 0.001**
Weekdays	750	87.7 ± 26.9	386	94.0 ± 27.3	364	80.9 ± 24.8	**< 0.001**
Weekend days	770	81.4 ± 29.8	394	87.1 ± 31.0	376	75.5 ± 27.4	**< 0.001**
Screen time (min/day)							
Overall	771	75.7 ± 37.4	396	77.0 ± 37.1	375	74.4 ± 37.7	0.33
Weekdays	777	62.7 ± 34.9	399	63.2 ± 34.9	378	62.2 ± 34.9	0.68
Weekend days	772	108.4 ± 62.4	396	111.7 ± 61.7	376	104.8 ± 63.0	0.12
Sleep duration (h:min/day)							
Overall	771	10:21 ± 0:33	395	10:19 ± 0:33	376	10:23 ± 0:33	0.054
Weekdays	777	10:17 ± 0:35	398	10:15 ± 0:35	379	10:19 ± 0:35	0.079
Weekend days	772	10:32 ± 0:42	396	10:29 ± 0:43	376	10:34 ± 0:42	0.14

*Abbreviations*: *BMI* Body mass index, *BMI-SDS* Body mass index standard deviation score, *SD* Standard deviation

^a^According to Saari et al. (2011) [16]

^b^According to Cole et al. (2012) [17]

^c^Less than bachelor’s degree included comprehensive, vocation, or high school; bachelor’s degree included bachelor’s degree or college; and more than bachelor’s degree included master’s degree or licentiate/doctor

^d^T-test for continuous variables and chi-square test for categorized variables. Differences with *P* < 0.05 were considered statistically significant (statistically significant values are bolded)

**Fig. 1:**
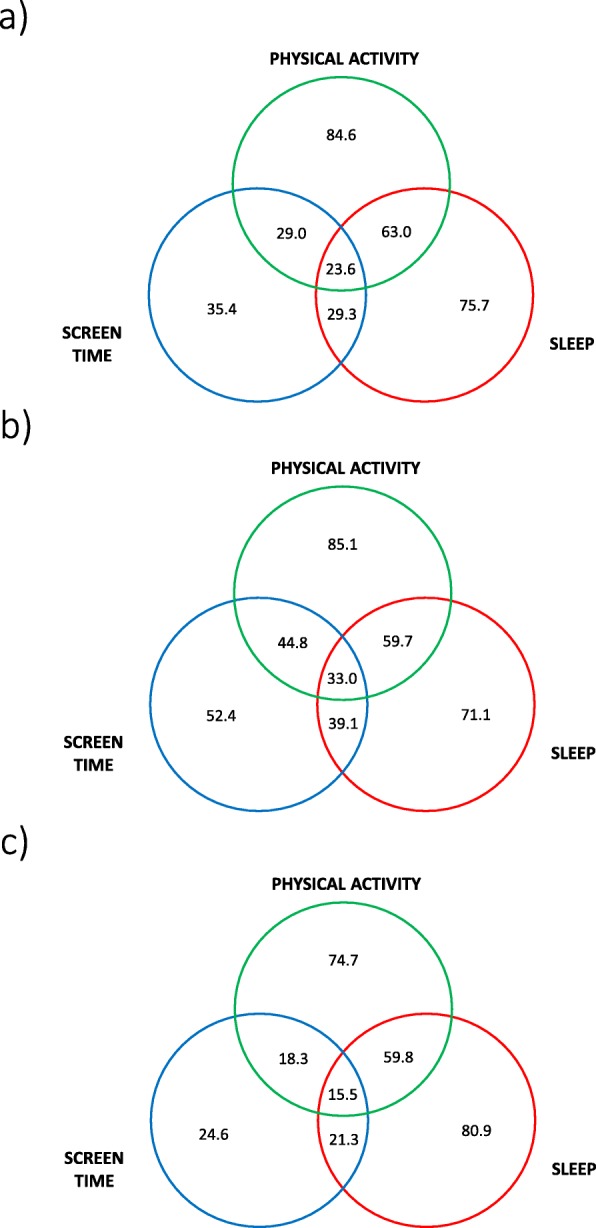
Compliance with the 24-h movement guidelines **a** overall (*N* = 721), **b** on weekdays (*N* = 727), and **c** on weekend days (*N* = 759)

A total of 23.6% of children complied with the 24-h movement guidelines overall (Fig. 1a), while the proportion was on weekdays 33.0% (Fig. 1b) and on weekend days 15.5% (Fig. 1c). The highest proportion of children met the PA guideline on weekdays (85.1%), while the proportion was slightly lower overall (84.6%) and on weekend days (74.7%). The highest proportion of children met the sleep guideline on weekend days (80.9%), followed by overall (75.7%) and on weekdays (71.1%). The compliance with meeting the guideline for ST was the highest on weekdays (52.4%), followed by overall (35.4%) and on weekend days (24.5%). No significant differences were found between boys and girls in terms of meeting the guidelines (data not shown).

Children who met the guidelines for PA overall, on weekdays, or on weekend days had a lower WC compared to children who did not meet the guidelines (Table 2). Moreover, children who met the guidelines for sleep overall and on weekend days had lower BMI and WC compared to children who did not meet the guidelines. Children who met the guidelines for both PA and sleep overall had lower BMI and WC, whereas meeting both guidelines on weekdays or on weekend days was associated with lower WC. There were no significant associations found between meeting all three guidelines (PA, ST, and sleep) and BMI or WC.

**Table 2:** Associations of meeting the recommendations for the 24-h movement guidelines with BMI and waist circumference

	BMI			Waist circumference		
	B (95% CI)	β	*p*	B (95% CI)	β	*p*
Physical activity						
Overall^a^	− 0.27 (− 0.57 to 0.02)	− 0.07	0.064	−1.37 (−2.04 to − 0.70)	− 0.13	**< 0.001**
Weekdays^b^	− 0.21 (− 0.50 to 0.09)	− 0.05	0.17	− 1.35 (− 2.02 to − 0.67)	− 0.12	**< 0.001**
Weekend^c^ day	0.01 (− 0.23 to 0.25)	0.00	0.93	− 0.61 (− 1.17 to − 0.05)	− 0.07	**0.033**
Screen time						
Overall	− 0.08 (− 0.29 to 0.13)	− 0.03	0.46	0.00 (− 0.49 to 0.50)	0.00	0.99
Weekdays	−0.06 (− 0.26 to 0.14)	−0.02	0.55	0.01 (− 0.46 to 0.48)	0.00	0.95
Weekend days	−0.04 (− 0.28 to 0.19)	−0.01	0.72	−0.05 (− 0.60 to 0.50)	−0.01	0.86
Sleep						
Overall	−0.26 (− 0.50 to − 0.03)	−0.09	**0.027**	−0.72 (− 1.26 to − 0.18)	−0.08	**0.009**
Weekdays	−0.14 (− 0.36 to 0.08)	− 0.05	0.22	−0.30 (− 0.81 to 0.22)	−0.03	0.25
Weekend days	−0.34 (− 0.60 to − 0.09)	−0.10	**0.008**	−0.79 (− 1.38 to − 0.19)	−0.08	**0.010**
Physical activity and screen time						
Overall	−0.16 (− 0.38 to 0.06)	−0.05	0.16	−0.28 (− 0.79 to 0.24)	−0.03	0.29
Weekdays	−0.11 (− 0.31 to 0.94)	−0.04	0.29	−0.33 (− 0.80 to 0.14)	−0.04	0.17
Weekend days	−0.07 (− 0.32 to 0.19)	−0.02	0.62	−0.32 (− 0.92 to 0.29)	−0.03	0.31
Physical activity and sleep						
Overall	−0.30 (− 0.52 to − 0.08)	−0.11	**0.007**	− 1.03 (− 1.53 to − 0.52)	−0.13	**< 0.001**
Weekdays	−0.18 (− 0.40 to 0.04)	−0.07	0.10	−0.70 (− 1.20 to − 0.20)	−0.09	**0.006**
Weekend days	−0.06 (− 0.27 to 0.15)	−0.02	0.60	−0.50 (− 0.99 to − 0.01)	−0.06	**0.044**
Screen time and sleep						
Overall	−0.06 (− 0.28 to 0.16)	−0.02	0.59	0.00 (−0.52 to 0.52)	0.00	1.00
Weekdays	−0.05 (− 0.25 to 0.16)	−0.02	0.67	0.10 (− 0.38 to 0.58)	0.01	0.68
Weekend days	−0.13 (− 0.37 to 0.12)	−0.04	0.32	−0.17 (− 0.75 to 0.41)	−0.02	0.57
Physical activity, screen time, and sleep						
Overall	−0.10 (− 0.34 to 0.14)	−0.03	0.41	−0.17 (− 0.72 to 0.39)	−0.02	0.56
Weekdays	−0.12 (− 0.34 to 0.10)	−0.04	0.28	−0.25 (− 0.75 to 0.25)	−0.03	0.34
Weekend days	−0.13 (− 0.40 to 0.15)	−0.03	0.38	−0.38 (−1.03 to 0.27)	− 0.04	0.25

Values are unstandardized (B) and standardized regression coefficients (β) with their *p*-values from linear regression analyses (statistically significant values are bolded). All models were adjusted for age, gender, season, and socioeconomic status (the highest education level in the family). Models including PA were additionally adjusted for awake wearing time of accelerometer, whereas models including waist circumference were additionally adjusted for height

*Abbreviations*: *BMI* Body mass index

^a^*N* = 694, ^b^*N* = 700, ^c^*N* = 715

Table 3 presents the means of BMI and WC [95% confidence intervals (CIs)] stratified by the number of participants meeting the guidelines. Meeting two or three guidelines overall compared to zero or one was associated with lower BMI and WC. There were no significant associations found between the number of guidelines met on weekdays or weekend days.

**Table 3:** Associations of the number of guidelines met with BMI and waist circumference

	Mean (95% CI)			Mean (95% CI) difference between groups		
	Meeting 0^a^ or 1^b^ guideline	Meeting 2^c^ guidelines	Meeting 3^d^ guidelines	2–1	3–1	3–2
Overall						
BMI	16.1 (15.9 to 16.3)	15.7 (15.6 to 15.8)	15.7 (15.5 to 15.9)	−0.39 (− 0.63 to − 0.15), ***p*** **= 0.002**	−0.38 (− 0.66 to − 0.10), ***p*** **= 0.008**	0.01 (− 0.24 to 0.26), *p* = 0.94
Waist circumference	54.5 (53.7 to 55.2)	53.2 (52.8 to 53.6)	53.5 (53.0 to 54.0)	−1.19 (− 1.73 to − 0.63), ***p*** **< 0.001**	−1.73 (− 1.68 to − 0.38), ***p*** **= 0.002**	0.16 (−0.41 to 0.73), *p* = 0.58
Weekdays						
BMI	15.9 (15.7 to 16.2)	15.8 (15.7 to 16.0)	15.7 (15.6 to 15.9)	−0.11 (− 0.37 to 0.15), *p* = 0.42	−0.20 (− 0.47 to 0.08), *p* = 0.16	−0.09 (− 0.32 to 0.15), *p* = 0.46
Waist circumference	54.0 (53.2 to 54.7)	53.5 (53.0 to 53.9)	53.6 (53.3 to 54.0)	−0.53 (−1.13 to 0.07), *p* = 0.082	− 0.63 (− 1.26 to 0.00), *p* = 0.05	−0.10 (− 0.63 to 0.44), *p* = 0.72
Weekend days						
BMI	15.9 (15.7 to 16.0)	15.8 (15.7 to 16.0)	15.7 (15.5 to 15.9)	−0.02 (− 0.24 to 0.21), *p* = 0.89	−0.17 (− 0.47 to 0.14), *p* = 0.29	−0.15 (− 0.44 to 0.14), *p* = 0.31
Waist circumference	53.9 (53.3 to 54.5)	53.7 (53.3 to 54.1)	53.0 (52.4 to 53.6)	−0.35 (− 0.88 to 0.18), *p* = 0.19	−0.67 (− 1.39 to 0.05), *p* = 0.069	−0.32 (− 0.99 to 0.36), *p* = 0.36

Linear regression models were adjusted for age, gender, season, and socioeconomic status (statistically significant values are bolded)

*Abbreviations*: *BMI* Body mass index, *CI* Confidence interval

^a^Overall *N* = 14, Weekdays *N* = 14, Weekend days *N* = 28

^b^Overall *N* = 173, Weekdays *N* = 149, Weekend days *N* = 212

^c^Overall *N* = 364, Weekdays *N* = 324, Weekend days *N* = 401

^d^Overall *N* = 170, Weekdays *N* = 240, Weekend days *N* = 118

## Discussion

The aims of the present study were to investigate the proportion of Finnish preschool children complying with the individual (i.e., PA, ST, and sleep) and combined 24-h movement guidelines for the early years as well as the association between complying with the guidelines and anthropometry. In accordance with the results, one-fourth of the preschool children met all three guidelines. Furthermore, meeting guidelines for PA and sleep were associated with lower BMI and WC, while there was no significant association observed regarding ST.

The proportion of children meeting all three guidelines within the 24-h movement guidelines was the highest on weekdays and the lowest on weekend days. This can partly be explained by the somewhat low compliance with the ST guideline. The majority of children met the guideline for PA overall and on weekdays, but on weekend days the highest compliance rate was for the sleep guideline. It is possible that parents might not have the same structure and routines to activate their children at home on weekends than the ECEC’s personnel do on weekdays. However, the results highlight the need to increase compliance, especially with ST. In addition, there should be more efforts put on promoting PA, particularly on weekend days, and sleep on weekdays.

There are only three previous studies that have been examined compliance with the 24-h movement guidelines for the early years in preschool children: one in Canada [8], one in Australia [9], and one in Belgium [10]. Similar to our study, the Canadian and Australian studies also reported the highest compliance rates for the PA and sleep guidelines, while the ST guideline received the lowest rates [8, 9]. In the Belgian study, however, the highest compliance was found for sleep and ST, while it was the lowest for PA [10]. The somewhat different findings can be partly explained by the methodological differences. For instance, in assessing PA the use of a different type of accelerometer device (ActiGraph vs. Actical), the use of different amounts of axes in capturing children’s acceleration (triaxial vs. uniaxial), and the use of a different cut-points in defining PA (Butte vs. Pate vs. Evenson) [8–10] can all affect the detected PA. This may further influence the proportion of children meeting the PA guideline. In addition, there were differences in assessing ST and sleep duration (open-ended questions vs. ready-given response categories), which may have affected the results. Due to these differences in methodology and study designs, comparing results to previous studies needs to be done with caution.

To the best of our knowledge, this is the first study that examines the associations of compliance with the 24-h movement guidelines for the early years with anthropometry in European preschool children. In accordance with our results, meeting the guidelines separately for PA or sleep, or for both of them, was associated with lower WC, whereas meeting the guidelines for sleep, or both PA and sleep, was associated with lower BMI. The Canadian study previously reported no significant associations between compliance with the 24-h movement guidelines and BMI in preschoolers [8], yet the differences in study designs need to be kept in mind. However, our results are in line with a large 12-country study in 9–11-year-old children (*N* = 6128) that investigated associations of compliance with the 24-h movement guidelines with BMI [18]. Although their guidelines were slightly different to the ones in younger children, they found that meeting all three guidelines regarding PA, ST, and sleep was associated with lower BMI. Moreover, we found that meeting two or three guidelines instead of none or one was associated with lower BMI and WC. These findings support the theory that these three movement behaviors are co-dependent, and thus, there is a great need for interventions that attempt to promote compliance of more than one movement behavior at a time.

Our results showed that the associations of meeting the guidelines for PA and/or sleep appeared to be stronger with WC compared to BMI. As an elevated BMI has been suggested to indicate more “overweight” than “over fatness” [19], WC has been commonly used as a marker for central obesity [20]. Thus, the findings indicate that meeting the guidelines for PA and sleep may have a role in decreasing visceral adipose tissue, which in turn has been related to adverse metabolic health consequences [20]. However, although we used accurate measures of anthropometry, we were not able to address associations between compliance with 24-h movement guidelines and body composition, such as fat mass or fat-free mass. Thus, in future studies, the use of more detailed measurements of body composition should be considered.

We found no significant association between meeting the guideline for ST with BMI or WC. It is possible that the rather short time, ≤60 min/day, was not enough to show the difference. Although ST has been connected to adverse health consequences, such as depressive symptoms and a poorer quality of health in children [21]**,** our finding is encouraging. The use of ST has also been considered a way to promote children’s digital technology skills as well as to enhance learning and creating competence in social interaction [22]. However, it is essential to further examine the associations between ST and different health outcomes, and to clarify, for instance, the role of ST duration in order to support children’s health.

### Strengths and limitations

The strengths of the present study include a relatively large sample of children as well as comprehensive assessments of PA, ST, and sleep duration. We used triaxial accelerometers instead of uniaxial in order to take into account children’s intermittent patterns of movement [13]. In addition, we chose to use the same cut-points that have also been used in other recent European studies on preschool children, increasing the comparability between the studies [23, 24]. The use of daily ST and sleep diaries with open questions instead of ready-given response categories in assessing ST and sleep were chosen to increase representativeness of ST and sleep. Finally, the daily ST diary included all types of ST (i.e., TV viewing, watching DVDs or videos, using tablets or smartphones, and using computers or playing computer games) instead of restricting it only to TV viewing [21].

The study also has some limitations that need to be considered. Firstly, the cross-sectional study design limits the conclusion about causality between the observed associations. Secondly, the sleep diary was filled in by the parents, and therefore, we have no information about whether the children had a nap while in ECECs. Thus, it is possible that the actual amount of sleep per day was slightly longer, which could increase the proportion of children meeting the guideline for sleep. Thirdly, we did not include any dietary factors (children’s energy intake) as we aimed to provide results that were well comparable with those of Chaput et al. [8]. Finally, due to the relatively low participation rate, the sample may be somewhat selected with participants interested in healthy lifestyles. This may have led to higher compliance rates than in the general population.

## Conclusions

Only one-fourth of the children met all three guidelines of the 24-h movement guidelines for the early years. The majority of preschool children met the guidelines for PA, followed by sleep. The lowest compliance was found for ST. Meeting recommendations for both PA and sleep was associated with a healthier weight status. However, there is still a need to improve compliance with the 24-h movement guidelines, especially for ST, in order to support health in young children.

## Data Availability

Not applicable.

## Abbreviations

BMI: Body mass index
BMI-SDS: Body mass index standard deviation score
CI: Confidence interval
ECEC: Early childhood education and care center
MVPA: Moderate to vigorous physical activity
PA: Physical activity
SD: Standard deviation
ST: Screen time
TPA: Total physical activity
WC: Waist circumference
